# Coronavirus Disease Model to Inform Transmission-Reducing Measures and Health System Preparedness, Australia

**DOI:** 10.3201/eid2612.202530

**Published:** 2020-12

**Authors:** Robert Moss, James Wood, Damien Brown, Freya M. Shearer, Andrew J. Black, Kathryn Glass, Allen C. Cheng, James M. McCaw, Jodie McVernon

**Affiliations:** The University of Melbourne, Melbourne, Victoria, Australia (R. Moss, F.M. Shearer, J.M. McCaw, J. McVernon);; University of New South Wales, Sydney, New South Wales, Australia (J. Wood);; The Peter Doherty Institute for Infection and Immunity at the University of Melbourne and Royal Melbourne Hospital, Melbourne (D. Brown, J.M. McCaw, J. McVernon);; University of Adelaide, Adelaide, South Australia, Australia (A.J. Black);; Australian National University, Canberra, New South Wales, Australia (K. Glass);; Monash University, Melbourne (A.C. Cheng);; Murdoch Children’s Research Institute, Melbourne (J. McVernon)

**Keywords:** respiratory infections, severe acute respiratory syndrome coronavirus 2, SARS-CoV-2, SARS, COVID-19, coronavirus disease, zoonoses, viruses, coronavirus, modelling, healthcare capacity, health sector capacity, isolation, quarantine, case-finding, contact tracing, ICU, ITU, social distancing, NPIs, nonpharmaceutical interventions, Australia

## Abstract

The ability of health systems to cope with coronavirus disease (COVID-19) cases is of major concern. In preparation, we used clinical pathway models to estimate healthcare requirements for COVID-19 patients in the context of broader public health measures in Australia. An age- and risk-stratified transmission model of COVID-19 demonstrated that an unmitigated epidemic would dramatically exceed the capacity of the health system of Australia over a prolonged period. Case isolation and contact quarantine alone are insufficient to constrain healthcare needs within feasible levels of expansion of health sector capacity. Overlaid social restrictions must be applied over the course of the epidemic to ensure systems do not become overwhelmed and essential health sector functions, including care of COVID-19 patients, can be maintained. Attention to the full pathway of clinical care is needed, along with ongoing strengthening of capacity.

As of late September 2020, >30.6 million confirmed cases of coronavirus disease (COVID-19) were reported worldwide, involving all global regions and resulting in >950,000 deaths (*1*). Although most cases are clinically mild or asymptomatic, early reports from China estimated that 20% of all COVID-19 patients progressed to severe disease and required hospitalization, 5%–16% of whom required management in an intensive care unit (ICU) (*2*). Pulmonary disease leading to respiratory failure has been the major cause of death in severe cases (*3*).

The ability of health systems around the world to cope with increasing case numbers is of major concern. All levels of the system will be challenged, from primary care, prehospital and emergency department (ED) services to inpatient units and ultimately ICUs. Stresses on clinical care provision will result in increased illness and death (*4*). Such tragic consequences already have been observed, even in high-income countries that provide the whole population with access to quality medical care. Greater effects can be expected in low- and middle-income countries where access to high-level care is extremely limited. Availability of ICU beds and ventilators has proven critical for the adequate management of severe cases, with overwhelming demand initiating complex ethical discussions about rationing of scarce resources (*5*).

To prepare for this challenge, Australia has drawn on approaches developed over many years to prepare for influenza pandemics (*6*), and rapidly produced a national COVID-19 pandemic plan (*7*). The plan reoriented relevant influenza pandemic response strategies toward this new pathogen, building on emerging understanding of its anticipated transmissibility and severity, which are the determinants of clinical impact (*8*). Early imposition of stringent border measures, high levels of testing, active case-finding, and quarantine of contacts all have bought time to reinforce public health and clinical capacity. However, an influx of cases among travelers returning from countries with rapidly growing epidemics have been associated with community transmission in several states in Australia. By April 14, 2020, a total of 6,366 cases and 61 deaths had been reported in the country (*9*).

We report on the use of a clinical care pathways model that represents the national capacity of the health system of Australia. This framework initially was developed for influenza pandemic preparedness (*10*) and has been modified to estimate healthcare requirements for COVID-19 patients and inform needed service expansion. The ability of different sectors to meet anticipated demand was assessed by modeling plausible COVID-19 epidemic scenarios, overlaid on available capacity and models of patient flow and care delivery. An unmitigated outbreak is anticipated to completely overwhelm the healthcare system in Australia. Given realistic limits on capacity expansion, these models have made the case for ongoing case-targeted measures, combined with broader social restrictions, to reduce transmission and flatten the curve of the local epidemic to preserve health sector continuity.

## Methods

### Disease Transmission Model

We developed an age- and risk-stratified transmission model of COVID-19 infection based on a susceptible-exposed-infected-recovered (SEIR) paradigm (Appendix). Transmission parameters were based on information synthesis from multiple sources, with an assumed basic reproduction number (R_0_) of 2.53 and a doubling time of 6.4 days (Table 1). Potential for presymptomatic transmission was assumed to be <48 hours before symptom onset. Despite an increasing body of evidence regarding requirements of hospitalized patients for critical care, considerable uncertainty remains regarding the full pyramid of mild and moderately symptomatic disease. Therefore, we simulated a range of scenarios by using Latin hypercube sampling from distributions in which the proportion of all infections severe enough to require hospitalization ranged from 4.3%–8.6%. These totals represent the aggregate of strongly age-skewed parameter assumptions (Table 2). For each scenario, corresponding distributions of mild cases being seen by primary care were sampled, ranging from 30%–45% at the lower range of the severe spectrum to 50%–75% for the most extreme cases and increasing linearly between the 2 ranges. Persons not seeking care in the healthcare system were assumed undetected cases without differentiation between those with mild or no symptoms.

**Table 1:** Parameter assumptions used in a coronavirus disease transmission model, Australia

Parameter	**Estimate or assumption**	**Justification**
Fundamental assumptions		
Doubling time	6.4 d	Estimated in from early case growth in Wuhan, China, from Wu et al. (*11*)
Incubation period	5.2 d	Based on Li et al. (*12*) and Lauer et al. (*13*)
Derived assumptions		
R_0_	2.53	Based on latent and infectious periods, with doubling time 6.4 d (Appendix)
Latent (noninfectious) period	3.2 d	Assumes 2 d of presymptomatic transmission before completion of incubation period, based on contribution estimates from Ganyani et al. (*14*) and Tindal et al. (*15*)
Infectious period	9.68 d	Estimated, related to doubling time and incubation period (Appendix)

**Table 2:** COVID-19 model severity parameter assumptions, relative to all denominator infections*

Age group, y	**% Hospitalized, range†**	**% Hospitalized in ICU, range‡**
0–9	0.03–0.06	0.01–0.02
10–19	0.03–0.06	0.01–0.02
20–29	0.39–0.78	0.11–0.23
30–39	1.4–2.90	0.43–0.85
40–49	2.55–5.11	0.75–1.50
50–59	4.95–9.90	1.45–2.91
60–69	7.75–15.49	2.27–4.55
70–79	17.88–35.76	5.25–10.50
>80	32.97–65.94	9.68–19.36
Mean bed-days	8 d	10 d
*COVID-19, coronavirus disease; ICU, intensive care unit. †Assumed proportional to ICU values and based on calibration to non–Hubei, China, severe case rates (Appendix). ‡Combines use of data from Intensive Care National Audit and Research Centre (*16***) **and COVID-19 Task Force of the Department of Infectious Diseases and Computer Service, Italy **(***17*), and assumptions used in Ferguson et al. (*18*). §Based on assumptions used in Ferguson et al. (*18*).		

### Case-Targeted Interventions

We simulated a case-targeted public health intervention. Cases were isolated at the point of diagnosis. We assumed isolation occurred 48 hours after symptom onset, limiting the effective infectious period and reducing infectiousness from the point of identification by 80%, enabling imperfect implementation. Targeted quarantine of close contacts was implemented in the model framework by dynamic assignment of a transient “contact” label. Each time a new infectious case appears in the model, a fixed number of temporary contacts are labeled. Only contacts can progress through the exposed and infectious states, however, most remain uninfected and return to their original noncontact status <72 hours. We assumed that 80% of identified contacts adhered to quarantine measures and that the overall infectiousness of truly exposed and infected contacts was halved by quarantine, given delayed and imperfect contact tracing and the risk for transmission to household members.

### Clinical Pathways Model

**Figure 1:**
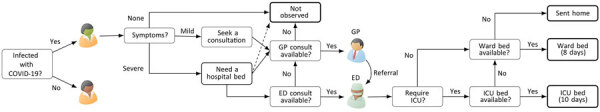
Clinical pathways model for used to assess national health system capacity for managing COVID-19 patients, Australia. The diagram demonstrates clinical pathways for mild and severe illness and assumes minor cases are managed within primary care. Unobserved patients are those who do not seek or are unable to access healthcare services. COVID-19, coronavirus disease; ED, emergency department; GP, general practitioner; ICU, intensive care unit.

At baseline of our clinical pathways model, we assume that half of available consulting and admission capacity across all healthcare sectors and services is available to COVID-19 patients. Mild cases are seen at primary care until capacity is exceeded. Severe cases access the hospital system through an ED and are triaged to a ward or ICU bed, if available, according to need. Requirements for critical care are assumed to increase steeply with age with the consequence that >60% of all infections requiring ICU admission occur in persons >70 years of age (Table 2). As ward beds reach capacity, the ability of EDs to adequately assess patients is reduced because of bed block, meaning that not all patients who need care are medically assessed, although some will still be able to access primary care. We assume that secondary infections are not affected by a person’s access to clinical care. The model allows for repeat patient visits within and between primary care and hospital services, and progression from ward to intensive care, with length of stay (Figure 1; Table 2). The model structure and assumptions are based on publicly available data on the healthcare system of Australia and expert elicitation (Appendix).

### Critical Care Capacity Expansion

The baseline assumption in our model was that half of currently available ICU beds would be available to COVID-19 patients. We considered 3 capacity expansion scenarios, assuming routine models of care for patient triage and assessment within the hospital system: total ICU capacity expansion to 150% of baseline, doubling the number of beds available to treat COVID-19 patients (2× ICU capacity); total ICU capacity expansion to 200% of baseline, tripling the number of beds available to treat COVID-19 patients (3× ICU capacity); or total ICU capacity expansion to 300% of baseline, increasing by 5-fold the number of beds available to treat COVID-19 patients (5× ICU capacity).

We also considered a theoretical alternative clinical pathway, COVID-19 clinics, which had constraints on bed numbers but double the capacity to assess severe cases in hospitals. The purpose of including this pathway was to reveal unmet clinical needs arising when bed block constrains ED triage capacity, potentially preventing needed admissions to the ICU.

### Social Distancing Interventions

Broad based social distancing measures overcome ongoing opportunities for transmission arising from imperfect ascertainment of all cases and contacts, and from presymptomatic and asymptomatic persons. In settings where nonpharmaceutical social interventions have been applied, associated case-targeted measures also have been in place, making the effectiveness of each difficult to quantify (*19*). Data from Hong Kong showing a reduction in influenza incidence arising from a combination of distancing measures introduced in response to COVID-19 provides good evidence of generalized transmission reduction (*20*). However, the relative quantitative contributions of different interventions, such as canceling mass gatherings, working remotely, closing schools, and ceasing nonessential services, cannot be differentiated reliably at this time (*18*).

Therefore, we focused on the overall objective of distancing, which is to reduce the reproduction number. We modeled the effect of constraining spread by 25% and 33%, overlaid on existing case-targeted interventions, which is consistent with observed impacts of combined measures less restrictive than total lockdown (*18*). These reductions in transmission equated to input reproduction numbers of 1.90 at 25% and 1.69 at 33%; the effective reproduction number in each scenario further was reduced by quarantine and isolation measures, which limit spread of established infection.

## Results

**Figure 2:**
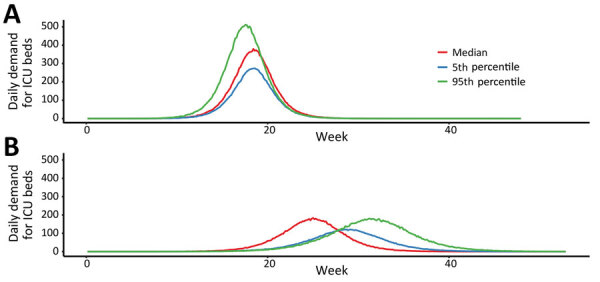
Estimated daily incidence of ICU admission demand per 1 million population during coronavirus disease (COVID-19) epidemic across all age groups, Australia. A) Demand during an unmitigated COVID-19 epidemic. B) COVID-19 epidemic mitigated by case-targeted public health measures. Lines represent single simulations based on median (red), 5th percentile (blue), or 95th percentile (green) final epidemic size. Of note, the more severe epidemic is more delayed by public health interventions due to a higher case proportion seeking medical attention. In a milder event, persons with non–medical seeking cases will continue to transmit in the community. This finding is contingent on the public health response capacity. ICU, intensive care unit.

According to our model, an unmitigated COVID-19 epidemic would dramatically exceed the capacity of the health system of Australia over a prolonged period (Figure 2). Case isolation and contact quarantine applied at the same level of effective coverage throughout the epidemic have the potential to substantially reduce transmission. By flattening the curve, these measures produce a prolonged epidemic with lower peak incidence and fewer overall infections (Figure 2). Epidemic scenarios with higher assumed severity, such as a 95th percentile case, are more effectively delayed by these public health measures than less severe scenarios, such as a 50th percentile case, because a higher proportion of all cases are seen by health services and can be identified for isolation and contact tracing. In a mitigated epidemic, overall use of the health system is increased because more patients are able to access needed care over the extended epidemic duration (Appendix Figure 3, panel A).

**Figure 3:**
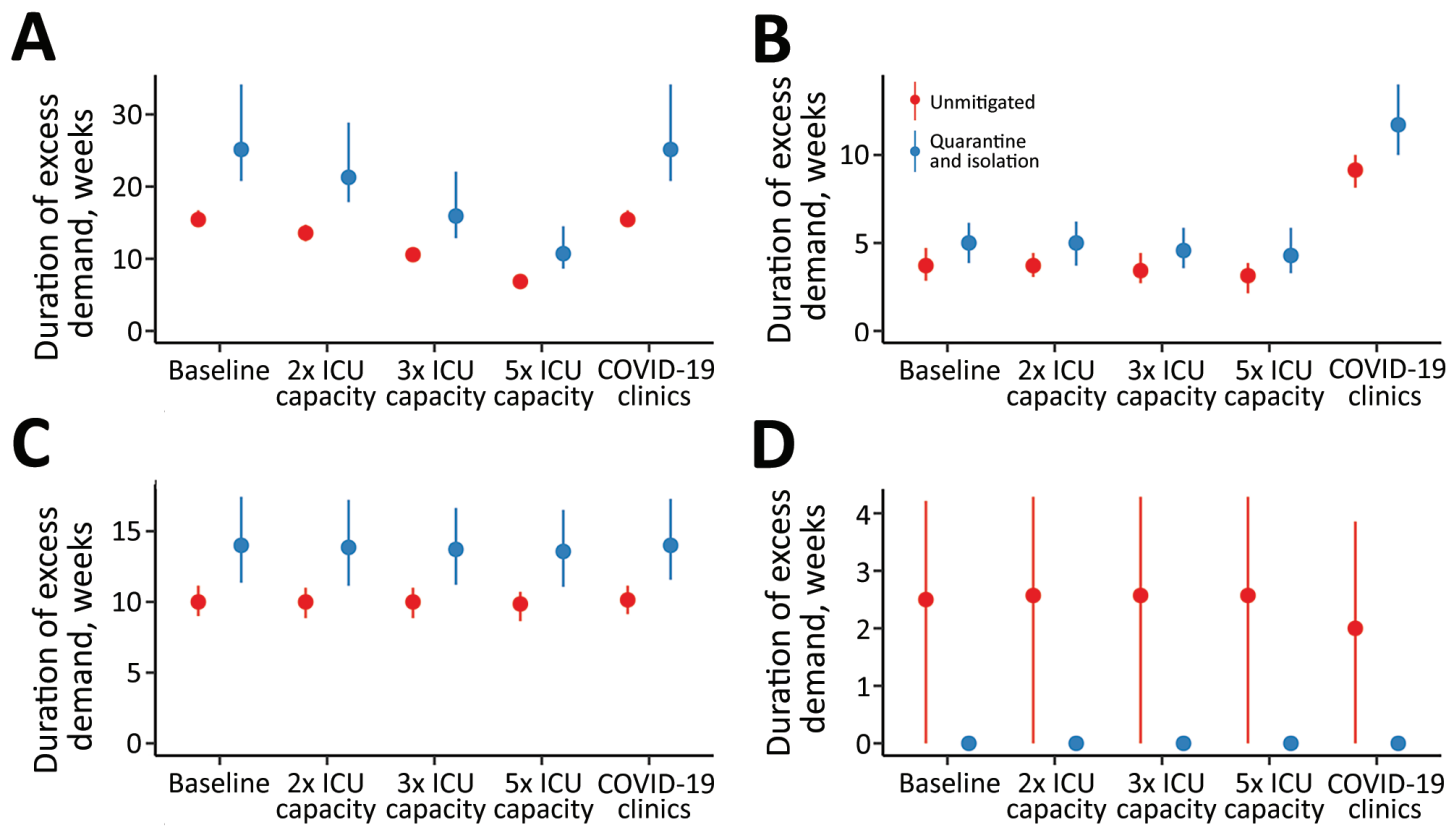
Estimated duration of excess demand for healthcare sector services during COVID-19 epidemic, Australia. The graphs compare exceedance for COVID-19 admissions for A) ICU beds; B) hospital ward beds; C) emergency departments; and D) general practitioner services at baseline, 2×, 3×, and 5× ICU capacity. The COVID-19 clinics scenario reflects an alternative triage pathway and baseline capacity. Red denotes unmitigated scenarios with no public health interventions in place; blue denotes the mitigated scenarios with quarantine and isolation in place. Dots denote the median; lines range from 5th–95th percentiles of simulations. COVID-19, coronavirus disease; ICU, intensive care unit.

**Figure 4:**
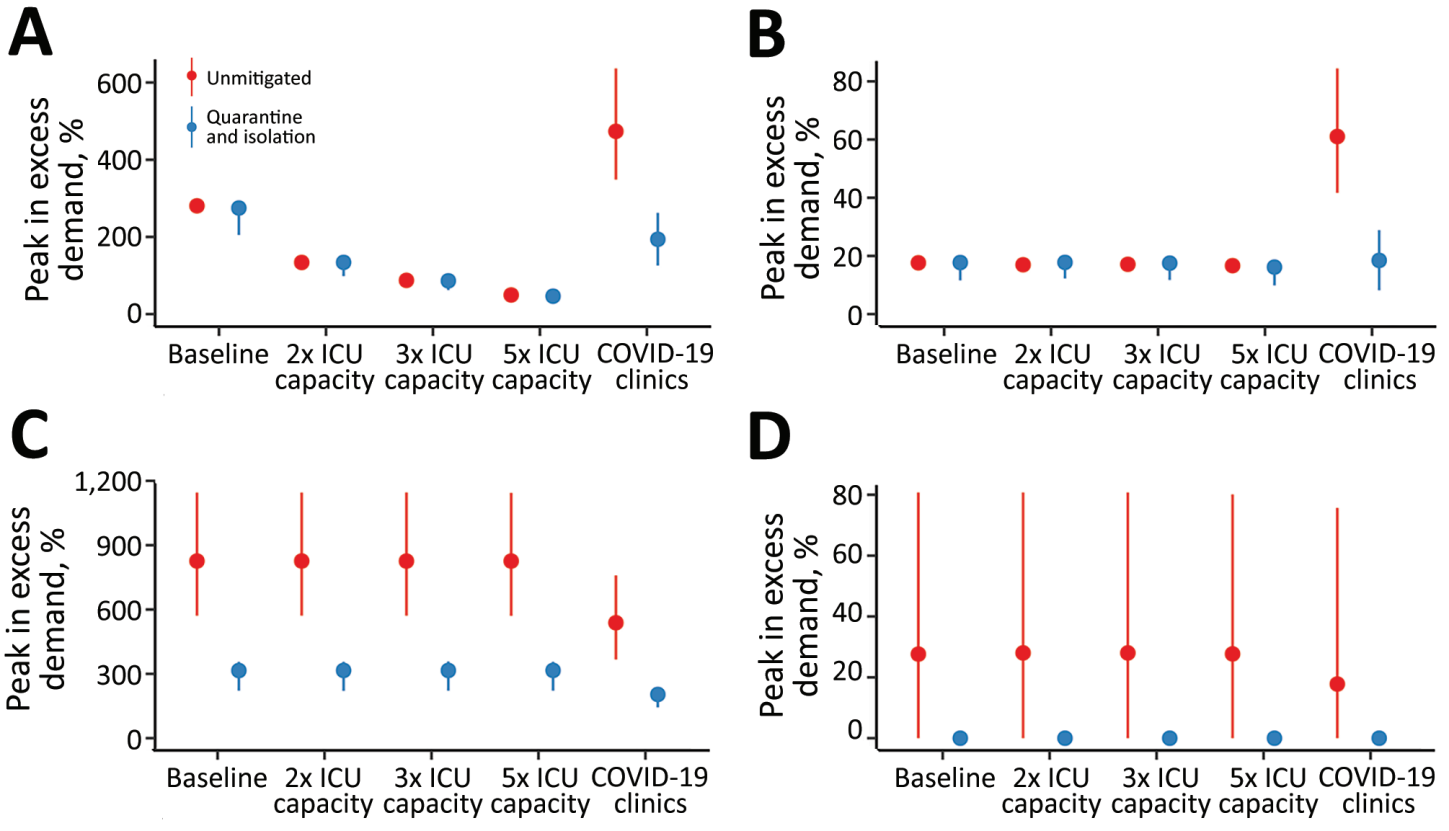
Estimated peak excess demand for healthcare sector services, by percentage, during the COVID-19 epidemic, Australia. The graphs compare exceedance for COVID-19 admissions for A) ICU beds; B) hospital ward beds; C) emergency departments; and D) general practitioner services at baseline, 2×, 3×, and 5× ICU capacity. The COVID-19 clinics scenario reflects an alternative triage pathway and baseline capacity. Red denotes unmitigated scenarios with no public health interventions in place; blue denotes the mitigated scenarios with quarantine and isolation in place. Dots denote the median; lines range from 5th–95th percentiles of simulations. COVID-19, coronavirus disease; ICU, intensive care unit.

Increasing the number of ICU beds available to patients with COVID-19 reduces the time over which ICU capacity is anticipated to be exceeded, potentially by more than half (Figure 3). The duration of exceedance for each capacity scenario is increased by quarantine and isolation because the overall epidemic is longer (Figure 3). During the period of exceedance, a degree of unmet need remains, even for the mitigated scenario (Figure 4). A 5-fold increase in the number of ICU beds available to patients with COVID-19 dramatically reduces the period and peak of excess demand (Figures 3, 4).

These figures do not accurately reflect the true requirement for services, however, because blocks in assessment pathways resulting from ED and ward overload are an upstream constraint on incident ICU admissions. The alternative triage scenario, the COVID-19 clinic, reveals a high level of unmet clinical need for both ward and critical care beds given baseline bed capacity (Figures 3, 4). Case-targeted measures overcame this limitation, to some extent, and effectively improved overall access to care (Figures 3, 4). Overall, if ICU beds available to COVID-19 patients are doubled, 10%–30% of those who require critical care receive it. The proportion rises to >20%–40% if capacity increases by 5-fold (Appendix Figure 3). These figures are quantified as total excess demand per million over the course of the epidemic (Appendix Figure 4).

**Figure 5:**
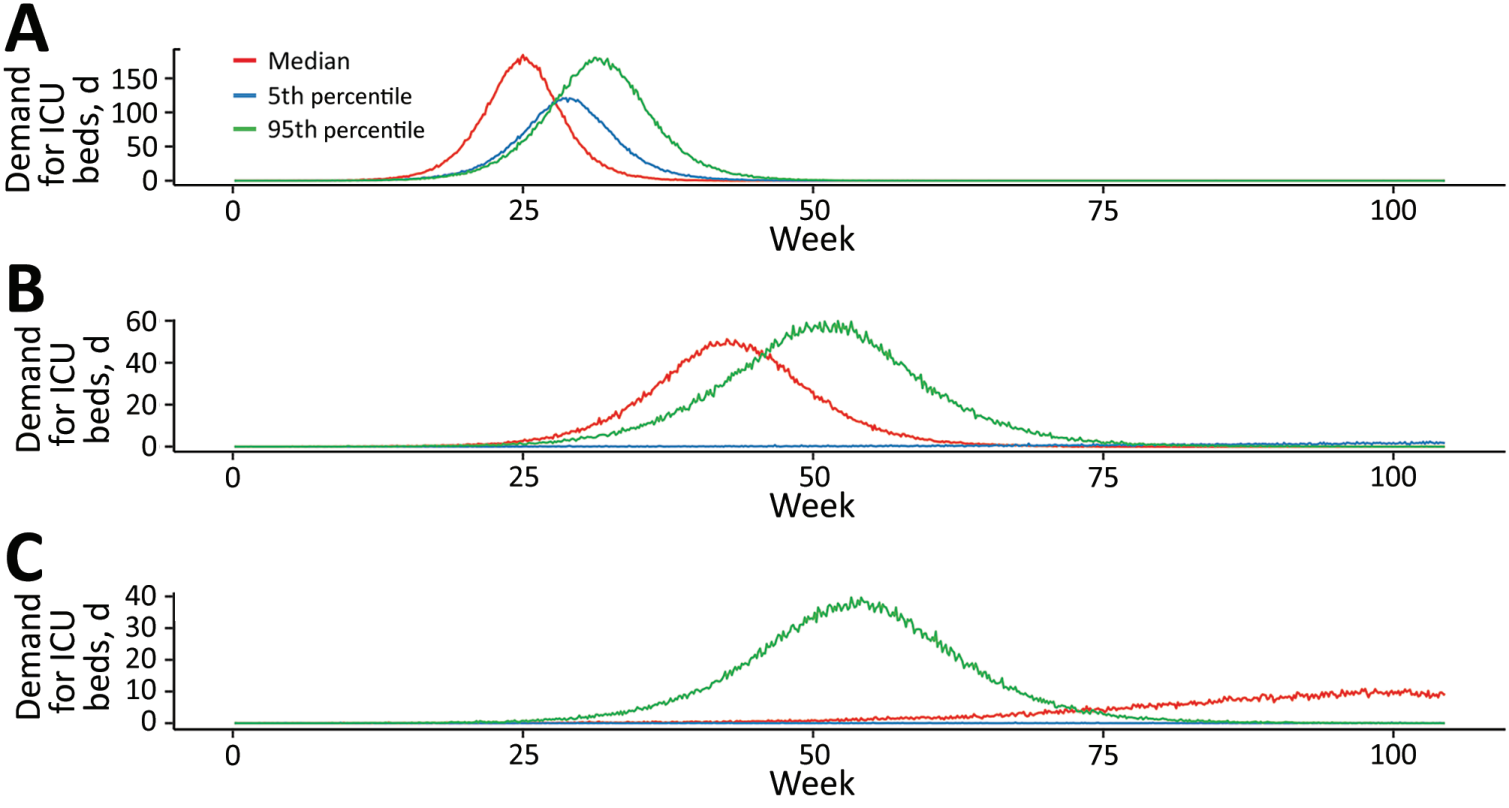
Estimated daily incident ICU admission demand per million population during coronavirus disease (COVID-19) epidemic, Australia. Comparison of mitigation achieved by A) quarantine and isolation alone; B) a further 25% mitigation due to social distancing; and C) a 33% mitigation. Lines represent single simulations based on median (red), 5th percentile (blue), or 95th percentile (green) parameter assumptions. ICU, intensive care unit.

Our simulated scenarios show that case isolation and contact quarantine alone will be insufficient to keep clinical requirements of COVID-19 cases within plausibly achievable expansion of health system capacity, even if very high and likely unrealistic levels of case finding can be maintained. We therefore explored the effects of additional social distancing measures that reduced input reproduction numbers by 25% and 33% on ICU requirements in relation to the same clinical care capacity constraints (Figure 5). Simulations assume ongoing application of measures of fixed effectiveness, which is also unlikely to be consistently achievable over an extended duration.

**Figure 6:**
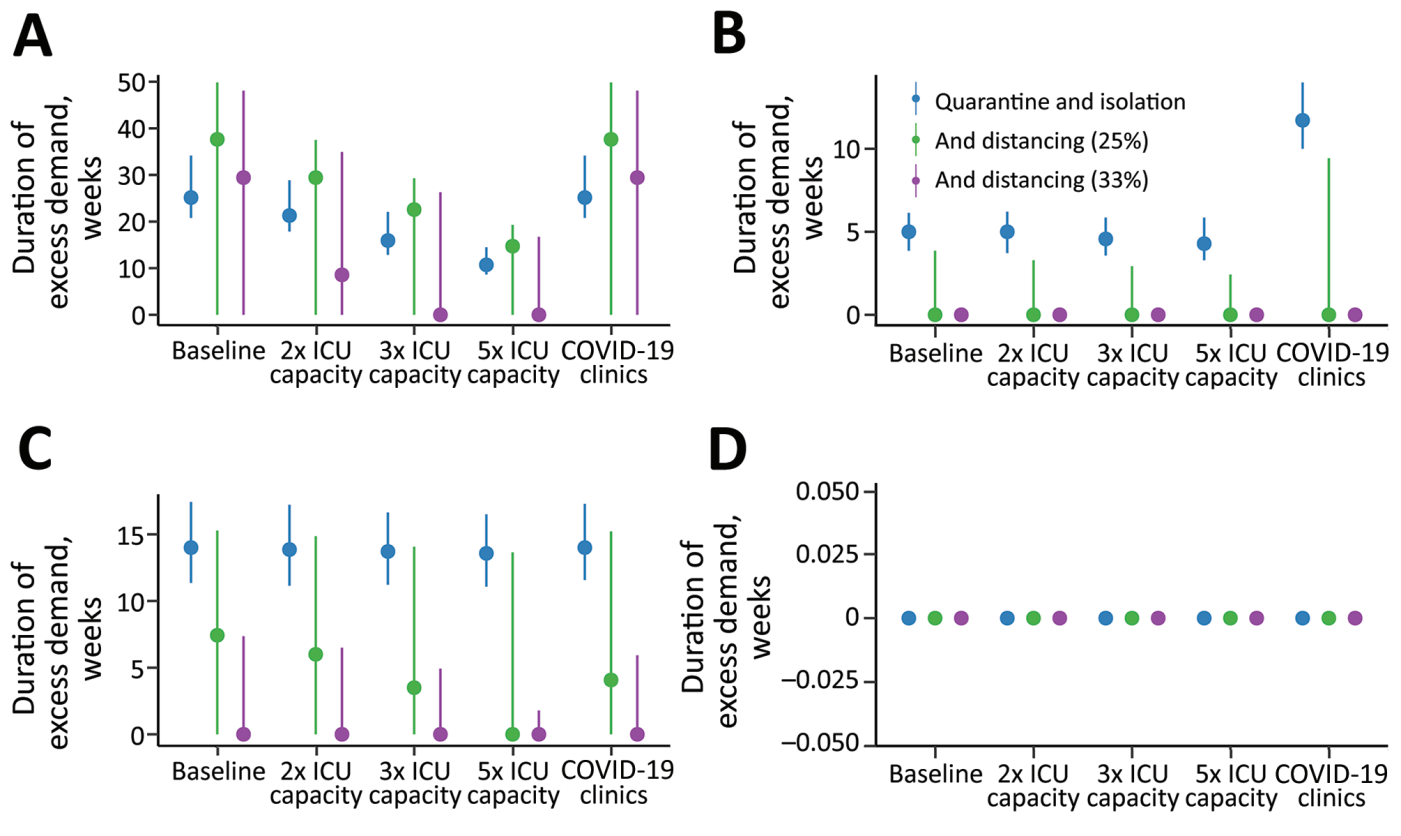
Estimated duration of excess demand for healthcare sector services compared with quarantine and isolation scenarios during the COVID-19 epidemic, Australia. The graphs compare exceedance for COVID-19 admissions for A) ICU beds; B) hospital ward beds; C) emergency departments; and D) general practitioner services at baseline, 2×, 3×, and 5× ICU capacity. Blue lines indicate quarantine and isolation only scenarios; green lines indicate overlaid social distancing measures that reduce transmission by an additional 25%; and purple lines indicate overlaid social distancing measures that reduce transmission by an additional 33%. The COVID-19 clinics scenario reflects an alternative triage pathway, and baseline capacity. Dots denote the median; lines range from 5th–95th percentiles of simulations. COVID-19, coronavirus disease; ICU, intensive care unit.

**Figure 7:**
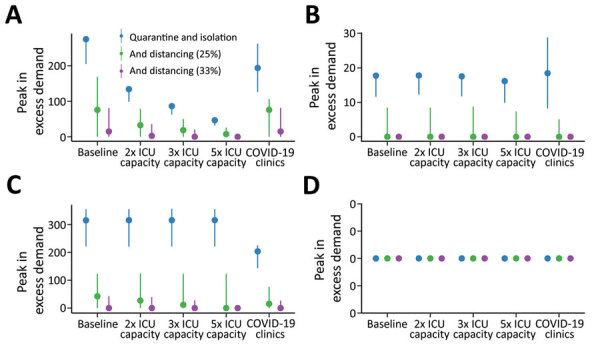
Estimated peak excess demand for healthcare sector services, expressed as percent available capacity, compared with quarantine and isolation scenarios during the COVID-19 epidemic, Australia. The graphs compare exceedance for COVID-19 admissions for A) ICU beds; B) hospital ward beds; C) emergency departments; and D) general practitioner services at baseline, 2×, 3×, and 5× ICU capacity. Blue lines indicate quarantine and isolation only scenarios; green lines indicate overlaid social distancing measures that reduce transmission by an additional 25%; and purple lines indicate overlaid social distancing measures that reduce transmission by an additional 33%. The COVID-19 clinics scenario reflects an alternative triage pathway, and baseline capacity. Dots denote the median; lines range from 5th–95th percentiles of simulations. COVID-19, coronavirus disease; ICU, intensive care unit.

The overlay of distancing measures, applied from the initial stages of the epidemic and maintained throughout, suppresses epidemic growth to a level that is within the range of plausible ICU capacity expansion. The duration of ICU exceedance remains long in the 25% case (Figure 6), but this overflow occurs to a far lesser degree than following case-targeted strategies only (Figure 7). As anticipated, a 33% reduction in transmission achieves greater benefits. Of note, pressure on ED consultations and ward beds also is eased substantially in these scenarios, maintaining capacity along the full pathway of care. As a result, the proportion of critical cases that can access care is greatly increased. Transmission reduction of 33% makes treatment for all cases achievable in most simulations if 3- to 5-fold ICU bed capacity can be achieved (Appendix Figure 3, panel B). This improvement is reflected in a large reduction in unmet need (Appendix Figure 4, panel B).

## Discussion

This modeling study shows that an unmitigated COVID-19 epidemic would rapidly overwhelm Australia’s health sector capacity. Case-targeted measures including isolation of those known to be infected, and quarantine of their close contacts, must remain an ongoing cornerstone of the public health response. These interventions effectively reduce transmission but are unlikely to be maintained throughout the epidemic course at the high coverage modeled here. As public health response capacity is exceeded, greater constraint of disease spread will be essential to ensure that feasible levels of expansion in available healthcare can maintain ongoing system functions, including care of COVID-19 patients. Broader based social and physical distancing measures reduce the number of potential contacts made by each case, minimizing public health workload and supporting sustainable case-targeted disease control efforts.

Our findings are consistent with a recently published model (*21*) that relates the clinical burden of COVID-19 cases to global health sector capacity, characterized at a high level. In unmitigated epidemics, demand rapidly outstrips supply, even in high-income settings, by a factor of 7 (*21*). Because hospital bed capacity is strongly correlated with income, this factor is greatly increased in low- and middle-income countries where underlying health status likely is poorer (*21*). Globally, marked variability in the definition of intensive care is observed, even in high-income countries where the descriptor covers many levels of ventilatory and other support. We concur with our conclusion that social distancing measures to suppress disease are required to save lives. In addition, we acknowledge that the marked social and economic consequences of such measures will limit their ongoing application, particularly in the settings where health systems are least able to cope with disease burden (*21*).

Much attention has been focused on expansion of available ICU beds per se, but our clinical model reveals that critical care admissions are further limited by the ability to adequately assess patients during times of system stress. In line with model recommendations, Australia, along with other countries, has implemented COVID-19 clinics as an initial assessment pathway to reduce impacts on primary care and ED services (*22*). Such facilities have additional benefits of ensuring appropriate testing, aligning local case definitions, and reducing the overall consumption of personal protective equipment by cohorting likely infectious patients. Evidence of bottlenecks as the epidemic progresses indicates that other measures to improve patient flows also should be considered, such as overflow expansion in EDs, encouraging and supporting home-based care, or early discharge to supported isolation facilities.

Quantitative findings from our model are limited by ongoing uncertainties about the true disease pyramid for COVID-19 and a lack of nuanced information about determinants of severe disease, which we represented by age as a best proxy. The clinical pathways model assumes that half of available bed capacity is available for patients with the disease but does not anticipate the seasonal surge in influenza admissions that might be overlaid with the epidemic peak, although even in our most recent severe season, 2017, only 6% of hospital beds were occupied by influenza cases (*23*). Available beds will likely be increased by other factors, such as secondary reductions in all respiratory infections and road trauma resulting from social restrictions, and purposive decisions to cancel nonessential surgery. Of note, we did not consider healthcare worker absenteeism due to illness, caregiving responsibilities, or burnout, all of which are anticipated challenges over a very prolonged epidemic accompanied by marked social disruption. We also cannot account for shortages in critical medical supplies because the true extent of these and their likely future impacts on service provision are currently unknown.

Our model indicates that a combination of case-targeted and social measures will need to be applied over an extended period to reduce the rate of epidemic growth. In reality, the stringency of imposed controls, their public acceptability, and compliance, likely will all vary over time. In Australia, compliance with isolation and self-quarantining was largely on the basis of trust in the early response during February–March, but active monitoring and enforcement of these public health measures is now occurring in many jurisdictions. Hong Kong and Singapore initiated electronic monitoring technologies from the outset to track the location of persons and enforce compliance (*24*). Proxy indicators of compliance, such as transport and mobile phone data, have informed understanding of the effect of social and movement restrictions on mobility and behavior in other settings (*19*), and will be further investigated in the context of Australia.

The effectiveness of multiple distancing measures, including lockdown, has been demonstrated in Europe, but the contributions of individual measures cannot yet be reliably differentiated (*18*). The effect of local measures to curb transmission will be estimated from real time data on epidemic growth in Australia, on the basis of multiple epidemiologic and clinical data streams. Estimates of the local effective reproduction number will enable forecasting of epidemic trajectories (*25*) to be fed into our analysis pathway. Anticipated case numbers will be used to assess the ability to remain within health system capacity represented by the clinical pathways model, given current levels of social intervention. Such evidence will support strengthening and, when appropriate, cautious relaxation of distancing measures. Further work will examine the effects of varying the intensity of measures over time, to inform the necessary conditions that would enable exit strategies from current stringent lockdown conditions to ensure maintenance of social and economic functioning over an extended time.

All these strategies, which combine to flatten the curve, will buy time for further health system strengthening and sourcing of needed supplies. Protecting the health and wellbeing of healthcare workers will be essential to ensure ongoing service provision. ICU capacity will need to be increased several-fold in anticipation of the looming rise in cases.

Multiple challenges must be overcome along the path to delivering safe and effective COVID-19 vaccines, and the timeframe for availability is highly uncertain (*26*). The search for effective therapies continues. Therefore, reducing COVID-19 illness and death relies on broadly applied public health measures to interrupt overall transmission, protect vulnerable groups, and maintain and strengthen the capacity of healthcare systems and workers to manage cases.

Appendix Additional information on design and formulas used for a coronavirus disease model to inform transmission reducing measures and health system preparedness, Australia.
