# Supplementary material for: Coronavirus Disease Model to Inform Transmission-Reducing Measures and Health System Preparedness, Australia
Source: Emerg Infect Dis. 2020 Dec;26(12):2844–53. doi: 10.3201/eid2612.202530 (PMC7706956; doi:10.3201/eid2612.202530)
Supplement: Appendix — Additional information on design and formulas used for a coronavirus disease model to inform transmission reducing measures and health system preparedness, Australia. [file 20-2530-Techapp-s1.pdf]

Article DOI: https://doi.org/10.3201/eid2612.202530

Coronavirus Disease Model to Inform Transmission Reducing Measures and Health System Preparedness, Australia

Appendix

Model Description

1. Epidemic Model Description

The model structure is shown in Appendix Figure 1. Model compartments are described in Appendix Table 1, model parameters are defined in Appendix Table 2, and population sub-groups are listed in Appendix Table 3.

2. Epidemic Scenarios

The compartmental model characterizing epidemic dynamics is defined by the following equations:

$$\frac{dS}{dt} = -\frac{S}{N} \cdot \lambda \quad [1]$$

$$\frac{dE_1}{dt} = \lambda\left(\frac{S}{N} - \rho\Theta_{\mathrm{M}}\right) - \sigma_1 E_1 \qquad \frac{dE_1^q}{dt} = \lambda\rho\Theta_{\mathrm{M}} - \sigma_1 E_1^q \quad [2]$$

$$\frac{dE_2}{dt} = \sigma_1 E_1 - \sigma_2 E_2 \qquad \frac{dE_2^q}{dt} = \sigma_1 E_1^q - \sigma_2 E_2^q \quad [3]$$

$$\frac{dI_1}{dt} = \sigma_2 E_2 - \gamma_1 I_1 \qquad \frac{dI_1^q}{dt} = \sigma_2 E_2^q - \gamma_1^q I_1^q \quad [4]$$

$$\frac{dI_2}{dt} = \gamma_1 I_1(1 - \alpha p_M) - \gamma_2 I_2 \qquad \frac{dI_2^q}{dt} = \gamma_1^q I_1^q(1 - \alpha p_M) - \gamma_2^q I_2^q \quad [5]$$

$$\frac{dR}{dt} = \gamma_2 I_2 \qquad \frac{dR^q}{dt} = \gamma_2^q I_2^q \quad [6]$$

$$\frac{dM}{dt} = \gamma_1 I_1(\alpha p_M) - \gamma_2 M \qquad \frac{dM^q}{dt} = \gamma_1^q I_1^q(\alpha p_M) - \gamma_2^q M^q \quad [7]$$

$$\frac{dR_M}{dt} = \gamma_2 M \qquad \frac{dR_M^q}{dt} = \gamma_2^q M^q \quad [8]$$

$$\alpha = \eta + \alpha_m (1 - \eta) \quad [9]$$

$$\beta = R_0 \times [(\gamma 1)^{-1} + (\gamma 2)^{-1}]^{-1} \quad [10]$$

$$\lambda = \lambda_{imp} + \beta(I_1 + I_2) + \beta \times (1 - Q_{eff})\,(I_1^q + I_2^q) + \beta \times (1 - M_{eff}) \times M + \beta_{M|Q} \times M^q \quad [11]$$

$$\beta_{M|Q} = \beta \times [1 - \max\,(M_{eff}, Q_{eff})] \quad [12]$$

$$\frac{dCT_M}{dt} = \kappa \times (\gamma_1 I_1 + \gamma_1^q I_1^q) \times (\alpha \times p_M) - \delta CT_M - \lambda \times \Theta_M \quad [13]$$

$$\frac{dCT_{NM}}{dt} = \kappa \times (\gamma_1 I_1 + \gamma_1^q I_1^q) \times (1 - \alpha \times p_M) - \delta CT_{NM} - \lambda \times \Theta_{NM} \quad [14]$$

$$\Theta_M = \frac{S}{N} \times \frac{CT_M}{CT_M + CT_{NM}} \quad [15]$$

$$\Theta_{NM} = \frac{S}{N} \times \frac{CT_{NM}}{CT_M + CT_{NM}} \quad [16]$$

2.1 Transmission assumptions

We based our transmission assumptions on initial estimates of a doubling time of 6.4 days and R_0 = 2.68 from Wuhan (*1*). In the initial version of this model, we assumed that all transmission occurred following an incubation period of 5.2 days, within a 2-stage infectious period of 7.68 days required to match the doubling time, R_0, and latent duration assumptions. However, as a result of increasing evidence of the importance of presymptomatic transmission (*2*,*3*), we have revised the latent period to 3.2 days to allow for 2 days of presymptomatic transmission. We elected to maintain the overall duration of infection and doubling time, which is consistent with a revised R_0 = 2.53. The 2-stage latent and infectious periods now have durations of 1.6 days each (latent period), and 4 and 5.68 days, respectively (infectious period). The associated generation interval for this parameterization is 6 days.

2.2 Mixing Assumptions

We stratified the Australian population by age (comprising 9 age groups) and by Indigenous status, to report hospitalization and ICU admission rates for each of these groups. We assumed homogeneous mixing across age groups and assumed that 80% of each Indigenous person's contacts were also Indigenous.

2.3 Intensive Care Unit (ICU) and Hospitalization Rates

As of February 12, 2020, ≈1,000 severe cases of COVID-19 had been reported outside Hubei Province, China (*4*). To establish an overall severe case-rate, we first extracted the number of cases outside Hubei, ≈11,340 cases reported on February 12, from the descriptive epidemiology publication from the Chinese Center for Disease Control and Prevention (China

CDC) (*5*), leading to an overall severe case rate of 8.8%. Because severity was not reported by age, we used other sources, in particular the recent Intensive Care National Audit & Research Centre report on 775 ICU admissions in the United Kingdom (*6*), to establish an appropriate age pattern. In brief, we extracted data on the proportion of ICU admissions by age and gender and then age and gender standardized these by using UK 2018 mid-year population figures (*7*), under the assumption that infection rates in adults are constant by age ≤70 years of age. These relative weightings after standardization and averaging over gender are 0.05 in persons 20–29 years of age, 0.19 in those 30–39 years of age, 0.33 in those 40–49 years of age, and 0.64 in those 50–59 years of age, compared with the reference group, persons 60–69 years of age. This enabled us to compute relative likelihoods of ICU admission by age in adults ≤70 years of age. We noted that male patients were substantially over-represented in this data, as reported in other settings but that substantially fewer persons ≥70 years of age were seen in healthcare facilities than expected, perhaps reflecting successful mitigation of transmission to these age-groups in the UK. Therefore, to establish appropriate baseline values in 60–69 years of age, 70–79 years of age, and ≥80 years of age we drew instead on the assumptions in Imperial College Report 9 (*8*) and then scaled values in younger adults by using the proportions described above. For children, we drew on the EpiCentro report of March 26 (*9*), in which 0/553 children with data available had been admitted to an ICU. Based on comparisons to notified incidence rates in persons >80 years of age, cases in persons <20 in Italy appear ≥30× underreported in comparison to population proportions. Scaling up by 30× and applying the rule of 3 (*10,11*), we estimated an upper bound on ICU risk as 1/5530 (≥0.018%), which we apply conservatively as our estimate in this age group.

To compute hospitalization rates by age, we extracted the age-distribution of cases outside of China from the China CDC report, and applied our ICU rates by age, scaled up by a constant factor to match the overall severe case rate of 8.8% from that setting. This exercise led to our assumption that 29% of hospitalized cases will require ICU care and is approximately equal to the proportion assumed in Imperial College Report 9 (*8*).

2.4 Range of scenarios

We considered the following 4 scenarios. We provide summary statistics for each scenario in Appendix Table 4. The following assumptions that apply across all 4 scenarios:

• The mean latent period is 3.2 days, the mean infectious period is 9.68 days, and the doubling time is 6.4 days.

• The baseline R_0 is 2.53, and the mean generation time is 6 days.

• Symptom onset occurs 2 days after the onset of infectiousness, so the mean incubation period is 5.2 days.

• Case ascertainment occurs 2 days after symptom onset.

• $\sigma_1 = \sigma_2 = 1.6$ days; $\gamma_1 = \gamma_1^q = 4.0$ days; $\gamma_2 = \gamma_2^q = 5.68$ days.

• All presenting cases can be isolated ($p_M = 1$).

• Imported cases arrive from overseas at a fixed, low rate ($\lambda_{imp} = 10$ cases per week).

The following assumptions differ between the 4 scenarios:

• There is no case isolation, or case isolation reduces transmission by 80% ($M_{\text{eff}} \in \{0,0.8\}$) from managed cases in M and M^q but has no effect on persons in I_1 or I_2.

• There is no self-quarantine (e.g., due to lack of contact tracing, or electing not to promote self-quarantine), or 80% of contacts will adhere to self-quarantine ($\rho \in \{0,0.8\}$).

• Self-quarantine halves transmission ($Q_{\text{eff}} = 0.5$) from persons in I_1^q and I_2^q.

• Physical distancing measures may reduce R_0 by 25% ($R = 1.8975$) or by 33% ($R = 1.6867$). We assumed these measures will be applied *in addition to* self-quarantine and case isolation.

The interventions considered in these scenarios, self-quarantine, case isolation, and physical distancing, are intended to represent broadly effective (but imperfect) public health measures and behavior changes in the population.

3. Models of Care

The structure of the clinical pathways model (Appendix Figure 2) is adapted from Moss et al. (*12*). Some infected persons will require hospitalization ("severe cases") and among the rest, some will present to outpatient settings ("mild cases"). The proportion of mild cases that

present to hospital EDs rather than to GP clinics in Australia was estimated to be 20%, based on expert consultation. We further assumed that a fraction of the severe cases will present to an outpatient setting early in their clinical course, in advance of requiring hospitalization. We assumed that a fixed fraction of hospitalized cases would require ICU admission. Parameters that govern these flows are listed in Appendix Table 5.

A key assumption of this clinical pathways model is that access to clinical care is independent of the infection process. Whether or not an infected person receives access to clinical care, they will give rise to the same number of secondary cases in the epidemic model. And the number of infected persons who receive clinical care is not related to the number of managed cases in the epidemic model (i.e., those who enter the M or M^q compartment). Case-finding and isolation as a public health response is considered separately from access to clinical care. In reality, public health response capacity may also be exceeded.

We assumed that a proportion of infected persons (α_s) will require hospitalization, and that this proportion varies by age. The upper bounds for each age group are listed in Appendix Table 3. A further proportion of infected persons (α_m) will present to outpatient settings but will not require hospitalization ("mild" cases). We introduce a scaling factor η from which we calculate α_s, and define the sampling distribution for this mild proportion, as per Moss et al. (*12*):

$$\eta_{\text{pow}} \sim U(\log_{10} 0.5, \log_{10} 1.0) \quad [17]$$

$$\eta = 10^{\eta_{\text{pow}}} \quad [18]$$

$$\alpha_m = \min(\alpha_m) + [\max(\alpha_m) - \min(\alpha_m)] \times \text{Beta}(\mu = 0.5, \text{Var} = 0.2) \quad [19]$$

$$\min(\alpha_m) = 0.05 + 0.2 \times \frac{\eta - 0.01}{0.99} \quad [20]$$

$$\max(\alpha_m) = 0.15 + 0.6 \times \frac{\eta - 0.01}{0.99} \quad [21]$$

$$\alpha_s = \eta \cdot \Pr(\text{Hosp}|\text{Inf}) \quad [22]$$

$$\alpha = \alpha_s + (1 - \alpha_s) \times \alpha_m \quad [23]$$

The lower and upper bounds for α_m are both linear functions of η. As the proportion of infected persons who require hospitalization increases, the proportion of infected persons who present to outpatient settings but not require hospitalization will increase, too.

National consultation and admission capacities for each healthcare setting were informed by public reports of healthcare infrastructure of Australia, under the assumption that, in a worst-case scenario, $\geq$50% of total capacity in each healthcare setting could possibly be devoted to COVID-19 patients (Appendix Table 6). Patients are admitted to general wards with a mean length of stay of 8 days and are admitted to ICUs with a mean length of stay of 10 days. Therefore, the prevalence of cases requiring hospitalization determines the available ward and ICU bed capacities for new admissions. At a jurisdictional level, daily presentations are allocated in proportion to each jurisdiction's resident population. Healthcare capacity is determined by the numbers of fulltime general practitioners (GPs) per jurisdiction, the yearly number of emergency department (ED) visits per jurisdiction, the number of overnight beds available in public hospitals by jurisdiction, and the number of intensive care unit (ICU) beds per jurisdiction, as described in the AIHW report, Hospital Resource 2017–18: Australian Hospital Statistics (*13*).

When the healthcare setting has insufficient capacity for a person to receive a consultation or to be admitted to hospital, the following steps are applied:

1. Severe cases that cannot receive an ED consultation (or a consultation with an alternate care pathway, if available) are not observed by the healthcare system and are reported as excess demand in this care setting.

2. Mild cases that cannot receive an ED or GP consultation (or a consultation with an alternate care pathway, if available) are not observed by the healthcare system and are reported as excess demand in this care setting.

3. Any severe cases that require ICU admission but cannot be admitted due to a lack of available ICU beds, are considered for admission to a general ward and are reported as excess ICU demand.

4. Any severe cases that cannot be admitted to a general ward due to a lack of available ward beds are observed by the healthcare system and are reported as excess ward demand.

3.1 Service Substitution Models

We consider a service-substitution model of care to circumvent EDs as the sole pathway for hospital admission.

COVID-19 Clinics for Triage and Hospital Admission

We assume that COVID-19 clinics are staffed by 10% of the GP and ED workforce, and that for each GP or ED consultation lost due to this decrease in staffing, 2 clinic consultations are gained. This is due to the assumption that every clinic consultation is allocated to a potential COVID-19 case, but only 50% of GP and ED consultations may be allocated to potential COVID-19 cases. When COVID-19 clinics are provided, we assumed that 25% of mild cases will use them in lieu of EDs and GPs, and that severe cases place equal demand on EDs and on COVID-19 clinics.

3.2 Critical care expansion

Recall that in the base care, COVID-19 patients have access to half of all ICU beds in the healthcare system. We consider three scenarios where ICU bed capacity is expanded:

Moderate: the number of ICU beds available to COVID-19 patients is doubled compared to the base, making 150% of total baseline ICU bed capacity available.

Large: the number of ICU beds available to COVID-19 patients is tripled compared with the base, making 200% of total baseline ICU bed capacity available.

Extreme: the number of ICU beds available to COVID-19 patients is increased 5-fold compared with the base, making 300% of total baseline ICU bed capacity available.

References

1. Wu JT, Leung K, Leung GM. Nowcasting and forecasting the potential domestic and international spread of the 2019-nCoV outbreak originating in Wuhan, China: a modelling study. Lancet. 2020;395:689–97. PubMed https://doi.org/10.1016/S0140-6736(20)30260-9
2. Ganyani T, Kremer C, Chen D, Torneri A, Faes C, Wallinga J, et al. Estimating the generation interval for COVID-19 based on symptom onset data. Euro Surveill. 2020;25:2000257. PubMed https://doi.org/10.2807/1560-7917.ES.2020.25.17.2000257

3. Tindale L, Coombe M, Stockdale J, Garlock E, Lau W, Saraswat M, et al. Transmission interval estimates suggest pre-symptomatic spread of COVID-19. Elife. 2020;9:e57149. PubMed https://doi.org/10.7554/eLife.57149

4. Chinese Center for Disease Control and Prevention. National Health Commission update; February 13, 2020 [cited 2020 Apr 14]. http://weekly.chinacdc.cn/news/TrackingtheEpidemic.htm#NHCFeb13

5. Novel Coronavirus Pneumonia Emergency Response Epidemiology Team. The epidemiological characteristics of an outbreak of 2019 novel coronavirus diseases (COVID-19) in China [in Chinese]. Zhonghua Liu Xing Bing Xue Za Zhi. 2020;41:145–51. PubMed https://doi.org/10.3760/cma.j.issn.0254-6450.2020.02.003

6. Intensive Care National Audit and Research Centre. INRC Case Mix Programme Database, Report on 775 patients critically ill with COVID-19. 2020 Mar 27 [cited 2020 Apr 14]. https://www.icnarc.org/About/Latest-News/2020/03/27/Report-On-775-Patients-Critically-Ill-With-Covid-19

7. Office for National Statistics. United Kingdom. Population estimates for the UK, England and Wales, Scotland and Northern Ireland: mid-2018. Technical report, 2019 [cited 2020 Apr 14] https://www.ons.gov.uk/peoplepopulationandcommunity/populationandmigration/populationestimates/bulletins/annualmidyearpopulationestimates/mid2018

8. Ferguson N, Laydon D, Nedjati-Gilani G, Imai N, Ainslie K, Baguelin M, et al.; Imperial College COVID-19 Response Team. Report 9: mpact of non-pharmaceutical interventions (NPIs) to reduce COVID-19 mortality and healthcare demand. Imperial College London; 2020 Mar 16. https://doi.org/10.25561/77482

9. COVID-19 Task Force of the Department of Infectious Diseases and Computer Service, Higher Institute of Health, Italy. Epidemiology of COVID-19, 2020 Mar 26 [in Italian] [cited 2020 Apr 14]. https://www.carditalia.com/epidemia-covid-19-aggiornamento-nazionale-iss-26-marzo-2020

10. Eypasch E, Lefering R. C K Kum, and Hans Troidl. Probability of adverse events that have not yet occurred: a statistical reminder. BMJ. 1995;311:619–20. PubMed https://doi.org/10.1136/bmj.311.7005.619

11. Ludbrook J, Lew MJ. Estimating the risk of rare complications: is the ‘rule of three’ good enough? ANZ J Surg. 2009;79:565–70. PubMed https://doi.org/10.1111/j.1445-2197.2009.04994.x

12. Moss R, McCaw JM, Cheng AC, Hurt AC, McVernon J. Reducing disease burden in an influenza pandemic by targeted delivery of neuraminidase inhibitors: mathematical models in the

Australian context. BMC Infect Dis. 2016;16:552. PubMed https://doi.org/10.1186/s12879-016-1866-7

13. Australian Government; Australian Institute of Health and Welfare. Hospital resources 2017–18: Australian hospital statistics 26 June 2019 [cited 2020 Apr 14]. https://www.aihw.gov.au/reports/hospitals/hospital-resources-2017–18-ahs

Appendix Table 1. Model compartments for the general population (middle column) and for persons who were quarantined as a result of contact tracing (right column)

Description	General	Quarantined
Susceptible persons	S	—
Latent period (first stage)	E_1	E_1^q
Latent period (second stage)	E_2	E_2^q
Infectious period (first stage)	I_1	I_1^q
Infectious period (second stage)	I_2	I_2^q
Recovered persons	R	R^q
Managed cases, ascertained upon leaving I_1 and less infectious than persons in I_2	M	M^q
Recovered persons that were managed cases	R	R_m^q
Contacts of unmanaged cases	CT_{NM}	
Contacts of managed cases, who will enter E_1^q if they become infected	CT_M	

Appendix Table 2. Model parameters

Symbol	Definition
σ_1	Inverse of first latent period.
σ_2	Inverse of second latent period.
γ_1	Inverse of first infectious period.
γ_2	Inverse of second infectious period.
γ_1^q	Inverse of first infectious period for quarantined cases.
γ_2^q	Inverse of second infectious period for quarantined cases.
η	Scaling factor for hospitalization proportion (“severe”).
α_m	Proportion of non-severe persons who present (“mild”).
α	Net proportion of persons who present.
R_0	The basic reproduction number.
λ	The net force of infection.
λ_{imp}	The force of infection from importation (10 exposures per week).
β	The force of infection exerted by one person.
κ	The per-person contact rate (20 persons per day).
δ	The duration of quarantine for contacts (14 d).
p_M	Probability of presenting cases being effectively managed†.
Q_{eff}	The reduction in infectiousness due to quarantine†.
M_{eff}	The reduction in infectiousness due to case management†.
ρ	The proportion of contacts (of ascertained cases) that will self-quarantine†.

†Key intervention parameters

Appendix Table 3. Population groups by age and Indigenous status, showing population sizes, and the probability of requiring hospitalization given infection*

Age	Indigenous	Non-Indigenous	Pr(Hosp/Inf), %†
0–9	184,560	2,966,400	0.062
10–18	149,040	2,466,480	0.062
19–29	151,440	3,651,120	0.775
30–39	93,360	3,315,360	2.900
40–49	87,360	3,154,560	5.106
50–59	66,960	2,964,720	9.895
60–69	38,880	2,397,120	15.493
70–79	15,360	1,423,440	35.762
80+	5,280	868,560	65.936

Age	Indigenous	Non-Indigenous	Pr(Hosp/Inf), %†

Demographic breakdown per Australian Bureau of Statistics resident population estimates, catalog number 3238.0.55.001, June 2016. The values of Pr(Hosp/Inf) are upper bounds; we defined the lower bounds to be half of these listed values.
†Probability of hospitalization given infection, by age (expressed as percentage).

Table 4. Key epidemic characteristics for each of the scenarios described above*

R†	Intervention	Attack Rate, %	Clinical AR, %	Hospital AR, %	Peak week
2.53	Unmitigated	89.1 (89.1–89.1)	37.9 (25.0–53.4)	5.4 (4.0–7.4)	18 (18–19)
2.53	Quarantine + isolation	67.5 (51.4–76.8)	28.6 (21.6–31.2)	4.0 (3.2–5.3)	30 (25–40)
1.90	Quarantine + isolation	37.7 (1.4–54.4)	15.5 (0.9–16.6)	2.2 (0.1–3.2)	58 (41–103)
1.69	Quarantine + isolation	11.6 (0.1–40.8)	5.0 (0.0–11.5)	0.8 (0.0–2.2)	85 (52–104)

*Median outcomes are reported, with 5th and 95th percentiles shown below in brackets. AR, attack rate.
†The effective reproduction number in the absence of self-quarantine and case isolation

Appendix Table 5. Parameters that characterize patient flows through the clinical pathways model*

Parameter	Value
Proportion of mild cases that present to GPs	80
Proportion of mild cases that present to EDs	20
Proportion of mild GP cases that revisit EDs	10
Proportion of mild ED cases that revisit GPs	5
Proportion of severe cases that present early	50
Proportion of early severe cases that present to GPs	80
Proportion of early severe cases that present to EDs	20
Proportion of non-early severe cases that present to EDs	100
Proportion of admitted cases that require ICU	29.335
Mean length of stay in ward beds, d	8 d
Mean length of stay in ICU beds, d	10 d
Ward bed availability threshold for reducing ED capacity	20
Minimum ED consultation capacity	10

*ED, emergency department; GP, general practitioner; ICU, intensive care unit.

Appendix Table 6. Estimated national and per-jurisdiction healthcare capacities, under the assumption that 50% of total capacity in each healthcare setting could possibly be devoted to COVID-19 patients*

Healthcare resource	National	ACT	NSW	NT	QLD	SA	TAS	VIC	WA
ICU beds	1,114	22	437	11	206	94	25	238	81
Ward beds	25,756	448	8,832	276	5,099	1,915	557	6,158	2,471
ED consultations	10,935	202	3,945	172	2,071	694	222	2,456	1,173
GP consultations	202,999	2,607	66,616	1,582	43,627	14,005	3,935	51,338	19,289

*ED and GP capacities reflect maximum number of daily consultations. ACT, Australian Capital Territory; ED, emergency department; GP, general practitioner; NSW, New South Wales; NT, Northern Territories; QLD, Queensland; SA, South Australia; TAS, Tasmania; VIC, Victoria; WA, Western Australia.

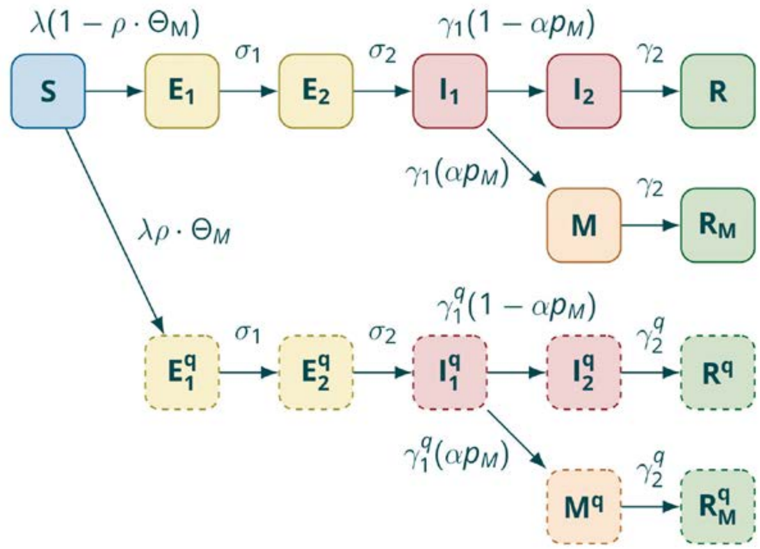

Appendix Figure 1. Model diagram. Some proportion p_M of presenting cases are ascertained and isolated. Quarantined persons (shown with dashed borders) exert a lesser force of infection than non-quarantined persons.

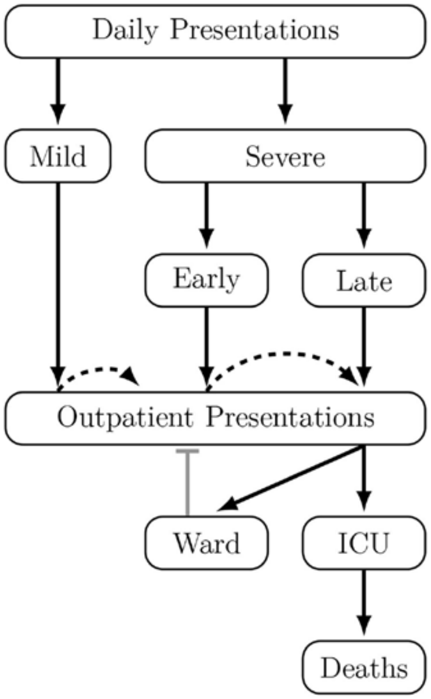

Appendix Figure 2. A schematic of the clinical pathways model. Repeat outpatient presentations are shown as dashed arrows. As ward bed occupancy increases, ED consultation capacity decreases (gray bar) and fewer severe cases can be triaged and admitted.

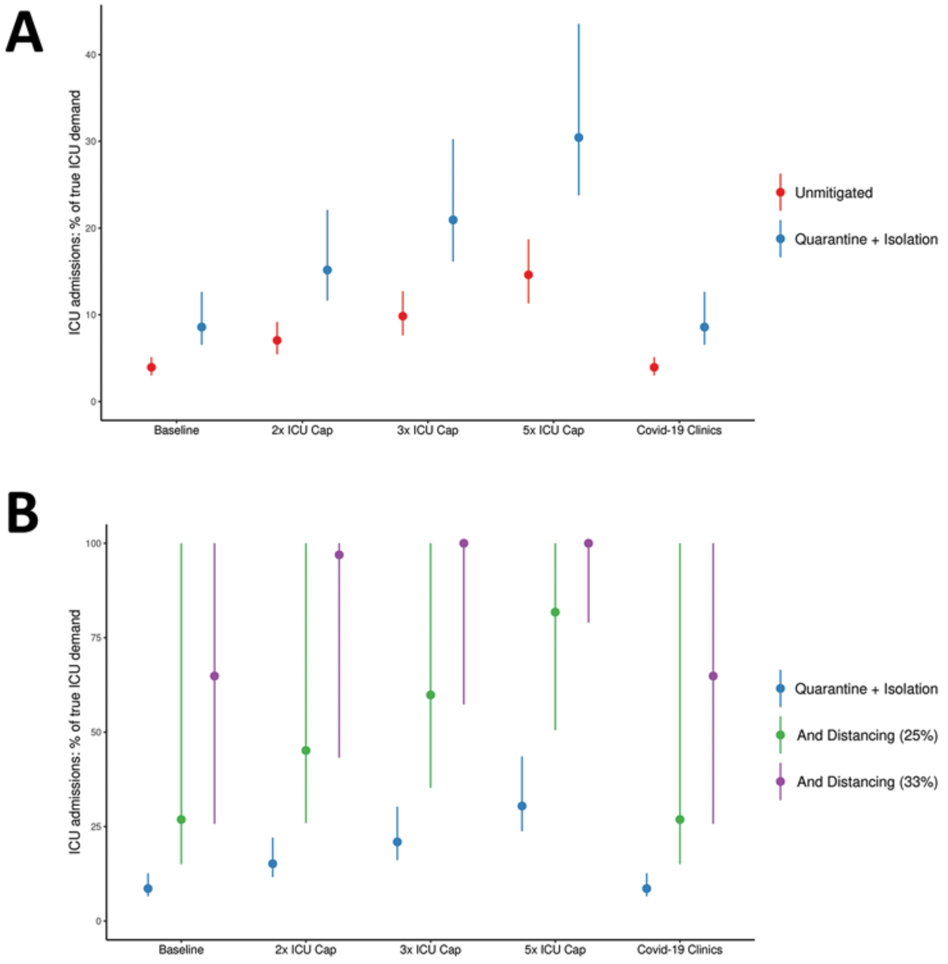

Appendix Figure 3. Estimated total intensive care unit (ICU) admissions throughout the course of the coronavirus disease (COVID-19) epidemic, as a percentage of true critical care demand, Australia. Scenarios shown are baseline, 2×, 3×, and 5× times ICU capacity for COVID-19 admissions. The COVID-19 clinics scenario reflects an alternative triage pathway, and baseline capacity. A) Red denotes unmitigated scenarios with no public health interventions in place; blue denotes the mitigated scenarios with quarantine and isolation in place. B) Blue denotes quarantine and isolation only scenarios; green denotes additional overlay of social distancing measures to reduce transmission by 25%; purple denotes additional overlay of social distancing measures to reduce transmission by 33%. Dots denote the median; lines range from 5th–95th percentiles of simulations.

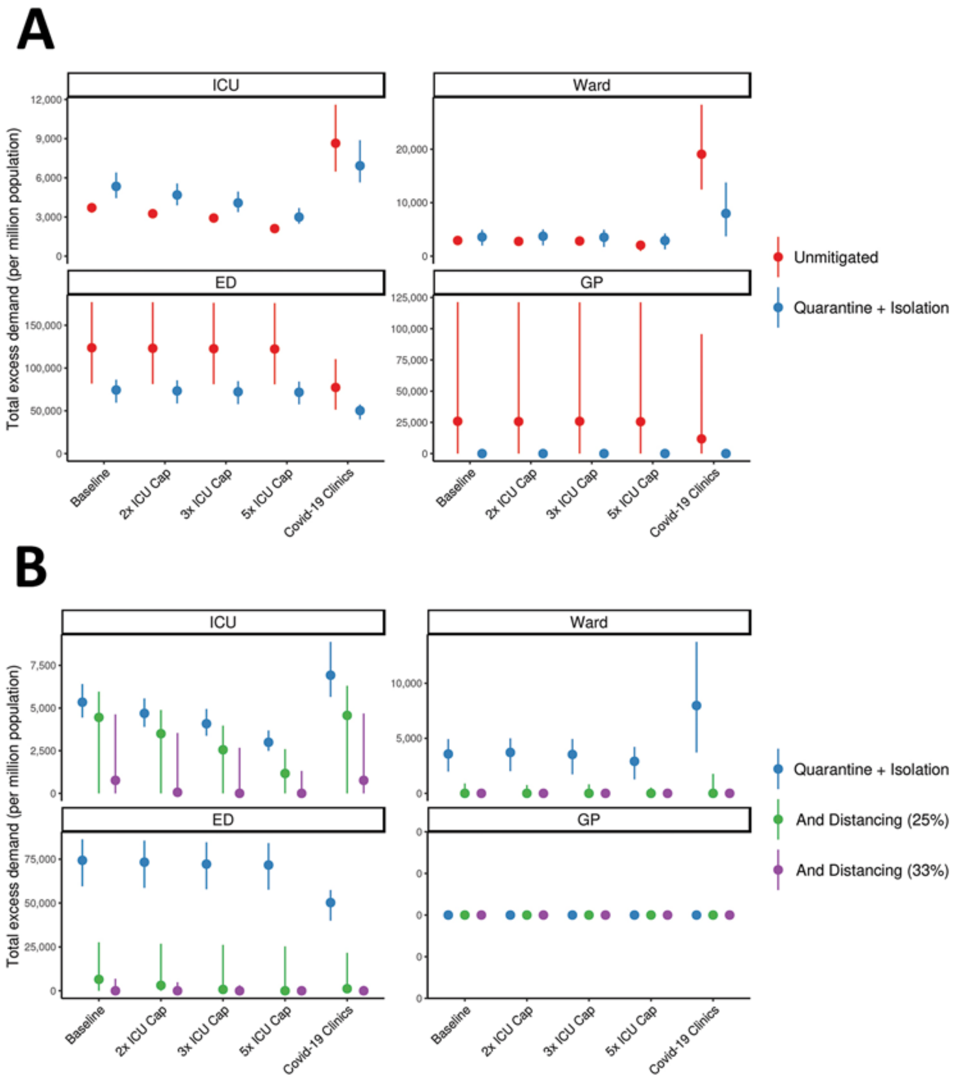

Appendix Figure 4. Total excess demand for services assessed by standard care pathways. Scenarios compared are baseline, 2×, 3×, and 5× times ICU capacity for COVID-19 admissions, and the alternative triage pathway (against baseline capacity). Dots denote the median; lines range from 5th–95th percentiles of simulations. A) Red denotes unmitigated scenarios; blue denotes quarantine and isolation scenarios. B) Blue denotes quarantine and isolation scenarios; green denotes additional overlay of social distancing measures to reduce transmission by 25%; and purple denotes additional overlay of social distancing measures to reduce transmission by 33%.
